# Physiological and transcriptomic analyses reveal mechanistic insight into the adaption of marine *Bacillus subtilis* C01 to alumina nanoparticles

**DOI:** 10.1038/srep29953

**Published:** 2016-07-21

**Authors:** Dashuai Mu, Xiuxia Yu, Zhenxing Xu, Zongjun Du, Guanjun Chen

**Affiliations:** 1 State Key Laboratory of Microbial Technology, Shandong University, Jinan 250100, PR China; 2 College of Marine Science, Shandong University (Weihai), Weihai 264209, PR China

## Abstract

An increasing number of studies have investigated the effects of nanoparticles (NPs) on microbial systems; however, few existing reports have focused on the defense mechanisms of bacteria against NPs. Whether secondary metabolism biosynthesis is a response to NP stress and contributes to the adaption of bacteria to NPs is unclear. Here, a significant induction in the surfactin production and biofilm formation were detected by adding Al_2_O_3_ NPs to the *B. subtilis* fermentation broth. Physiological analysis showed that Al_2_O_3_ NP stress could also affect the cell and colony morphogenesis and inhibit the motility and sporulation. Exogenously adding commercial surfactin restored the swarming motility. Additionally, a suite of toxicity assays analyzing membrane damage, cellular ROS generation, electron transport activity and membrane potential was used to determine the molecular mechanisms of toxicity of Al_2_O_3_ NPs. Furthermore, whole transcriptomic analysis was used to elucidate the mechanisms of *B. subtilis* adaption to Al_2_O_3_ NPs. These results revealed several mechanisms by which marine *B. subtilis* C01 adapt to Al_2_O_3_ NPs. Additionally, this study broadens the applications of nanomaterials and describes the important effects on secondary metabolism and multicellularity regulation by using Al_2_O_3_ NPs or other nano-products.

There has been a quantum increase in the use of nanoparticles (NPs) in many spheres of life. The physical and chemical properties of NPs can vary significantly from those of their bulk counterparts^1^. Nanoparticles are being considered for use in combating diseases such as cancer^2^, or fighting bacterial pathogens^3^. Beyond biomedical applications, there are established uses of nanoparticles for industrial applications and commercial products.

The increased presence of NPs in environment necessitates a basic understanding of their interactions with biomolecules and biological systems. The toxic effects of nanoparticles, termed “nanotoxicity,” are increasingly evident. Previous studies in animals and cell culture have amply demonstrated loss of cell viability, tissue damage and inflammatory reactions^4^.

Recently, an increasing number of studies have investigated the effects of NPs on microbial systems. The antimicrobial properties of NPs are attractive for their efficacy and low cost, and they have been demonstrated against a wide range of microorganisms, including drug-resistant strains^5^. Nanoparticles have been shown to inhibit growth of *Escherichia coli*, *Pseudomonas aeruginosa*, *Klebsiella pneumonia*, and several other multidrug-resistant microorganisms^6^. Zinc oxide and magnesium oxide NPs were found to exert significant growth inhibitory effects, which were related to membrane damage and oxidative stress responses in *Escherichia. coli*^7^. Nitric-oxide-releasing NPs are able to change the structure of the bacterial membrane and produce reactive nitrogen species (RNS), which lead to modification of essential bacterial proteins^8^. In contrast, Ag NPs and Cu NPs prevent biofilm formation, induce ROS generation, and cause DNA damage in common pathogens, such as *E. coli*^9^,^10^. It should be mentioned that Al_2_O_3_ NPs could attach to the cell wall and travel into the cytoplasm of *E. coli*, where they exert toxic effects^11^,^12^. Also, Al_2_O_3_ NPs could cause the cell wall damage and lipid peroxidation then caused a decrease in cell viability of *Bacillus licheniformis*^12^. However, these reports mainly focused on the antibacterial properties of NPs, whether or how microorganism adapt to NP stress remains unclear.

*Bacillus* species as biological control agents are receiving increased attention because of their ability to produce various antimicrobial substances. Additionally, these species are commonly used as a model Gram-positive strain for drug-resistance analysis. As a result, the antimicrobial effects of NPs have been explored with *B. subtilis*. Toxicity of Ag and ZnO NPs towards *B. subtilis* is significantly less due to the presence of a thicker peptidoglycan layer^13^. Previous studies have addressed the role of a limited sub-set of *B. subtilis* genes in response to Al_2_O_3_ NPs but the potentially pan-metabolic action of Al_2_O_3_ NPs on cells alludes to large-scale genetic regulation^14^. For Al_2_O_3_ NPs, the toxic mechanism may be enhanced by association of the nanoparticle and bacterial surface and subsequent cell wall binding followed by the enhancement of permeability^15^, however, how *B. subtilis* adapt to the Al_2_O_3_ NPs remains unknown. In our earlier study, we reported that Al_2_O_3_ NPs can be used as effective flocculants for flocculating *B. subtilis*, and the possible attachment mechanisms of Al_2_O_3_ NPs to the *B. subtilis* surface may be electrostatic^16^. Whether this electrostatic attachment could affect or change the physiological phenotype and development or affect secondary metabolism remained unclear.

Nearly 30 years ago, James A. Shapiro proposed multicellularity as a general bacterial trait^17^, and *B. subtilis* is now one of the classical and best-studied bacterial species^18^. Given that Al_2_O_3_ NPs damage the bacterial cell wall and increase permeability, resulting in growth inhibition, we wondered whether or how Al_2_O_3_ NPs affect multicellularity and secondary metabolism of *B. subtilis*, and how *B. subtilis* adapt to a certain concentration of Al_2_O_3_ NPs. To test this aim, various concentrations of Al_2_O_3_ NPs were added during the culturing and fermentation of surfactin of *B. subtilis*. We noted a significant induction in the surfactin production and biofilm formation by adding Al_2_O_3_ NPs in the fermentation broth. Al_2_O_3_ NPs also influenced the motility, colony morphology, and sporulation. Furthermore, a suite of toxicity assays testing membrane damage, cellular ROS generation, electron transport activity and membrane potential was used to determine the molecular mechanisms of toxicity of Al_2_O_3_ NPs compared to their microsized analogues. To capture the overall genetic response to Al_2_O_3_ NPs and bulk-Al_2_O_3_ and to explore the mechanism of adaption to Al_2_O_3_ NPs, whole transcriptomic analysis was used. These results reveal a new mechanism of how marine *B. subtilis* C01 adapted to alumina NPs. Additionally, this study broadens the potential applications of nanomaterials and has important implications for secondary metabolism and multicellularity regulation by using Al_2_O_3_ NPs and for exploring other nano-products useful in product fermentation or bio-medical applications.

## Results

### Effect of Al_2_O_3_ NPs on biofilm formation

**Figure 1:**
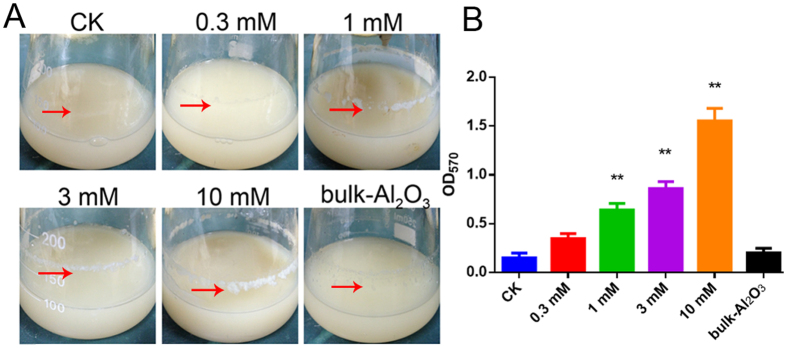
Phenotypic analysis and quantification of biofilm formation. CK means control, 0.3 mM, 1 mM, 3 mM, 10 mM mean various concentration of Al_2_O_3_ NPs, bulk-Al_2_O_3_ means 10 mM bulk-Al_2_O_3_. (**A**) Phenotypic analysis of biofilm formation on the flask, the fermentation broth was 100 ml in the 250 ml flask, treated with different concentration of Al_2_O_3_ NPs for shaken culturing 60 h. The white cycles on the flask was the biofilms; (**B**) Quantification of biofilm by staining with crystal violet (**p < 0.01, n = 3).

**Figure 2:**
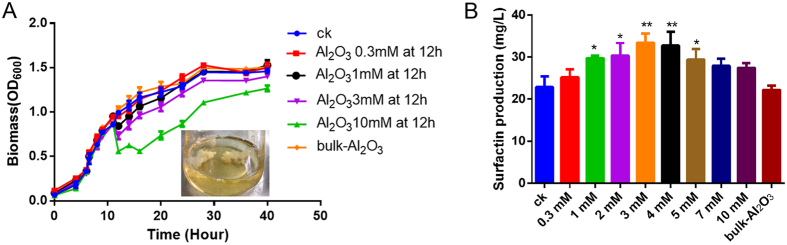
Al_2_O_3_ NPs enhances the surfactin production. (**A**) Time course of *B. subtilis* growth and the dosage of Al_2_O_3_ NPs treatment on fermentation; (**B**) Surfactin production of *B. subtilis* treated with various concentration of Al_2_O_3_ NPs, Methanolic extracts containing surfactin from cell-free supernatants of various treatment were fractionated by RP-HPLC analysis and detection at 214 nm. CK means control, 0.3 mM, 1 mM, 2 mM, 3 mM, 4 mM, 5 mM, 7 mM, 10 mM mean various concentration of Al_2_O_3_ NPs, bulk-Al_2_O_3_ means 10 mM bulk-Al_2_O_3_ (*P < 0.05, **P < 0.01, n = 3).

In our previous study, it was reported that Al_2_O_3_ NPs can be used as effective flocculants for flocculation of *B. subtilis*^16^. During the flocculation by using different concentrations of 40 nm Al_2_O_3_ NPs, which have been characterised in our earlier study^15^, biofilm formation was also found to be influenced to varying degrees (Fig. 1A). After treating *B. subtilis* with 0.3, 1, 3, or 10 mM of 40 nm Al_2_O_3_ NPs and continuing shake culturing for 60 h, biofilm formation of *B. subtilis* was enhanced as the concentration of Al_2_O_3_ NPs increased, although high concentrations of Al_2_O_3_ NPs could inhibit the growth of planktonic cells (Figs 1B and 2A). The quantitative analysis of biofilm formation using crystal violet was similar to the phenotypic analysis (Fig. 1A,B).

However, it remained unknown whether Al_2_O_3_ NPs had the same effect on *B. subtilis* when stationary culturing. To test this, biofilm formation was monitored when Al_2_O_3_ NPs were added in the liquid fermentation broth followed by stationary culturing. In contrast to shake culturing, Al_2_O_3_ NPs prevented biofilm formation in stationary culturing (Fig. S1), which was probably due to the flocculation effect of Al_2_O_3_ NPs (Fig. 2A), which resulted in the restriction of motility. However, the exact mechanisms need to be determined in subsequent studies. Taken together, Al_2_O_3_ NPs appear to be involved in the regulation of biofilm formation.

### Effect of Al_2_O_3_ NPs on surfactin production

Surfactin was quantified using HPLC to determine whether the surfactin production changed after the flocculation by Al_2_O_3_ NPs. The results showed 3 mM Al_2_O_3_ NPs could induce the surfactin production (Fig. 2B). High concentrations of Al_2_O_3_ NPs inhibit the growth of microorganisms^15^; therefore, organisms require a suitable concentration and induction time of Al_2_O_3_ NPs to adjust the growth and metabolism. To evaluate this effect, the concentration of Al_2_O_3_ NPs was varied from 0 to 10 mM. Surfactin accumulation increased with increasing concentrations of Al_2_O_3_ NPs until a limiting maximum concentration (3 mM or 4 mM) was reached (Fig. 2A,B), and growth was reduced when the dosage was 10 mM. In response to varying Al_2_O_3_ NPs induction times (0–72 h), the highest yield (33.5 mg/L) was at 12 h (Fig. S2). It is well knows that NP size holds an intriguing role on its physico-chemical property and subsequent effect on microbial system. In our study, we found that 3 mM small size (40 nm) of Al_2_O_3_ NPs could induce more surfactin production compared with that induced by large size (110 nm and 280 nm) of Al_2_O_3_ NPs (data not shown). As a result, the addition of 3 mM Al_2_O_3_ NPs (40 nm) at 12 h to the fermentation media is the suggested treatment condition for further physiological study.

### Al_2_O_3_ NPs influence morphogenesis and motility

**Figure 3:**
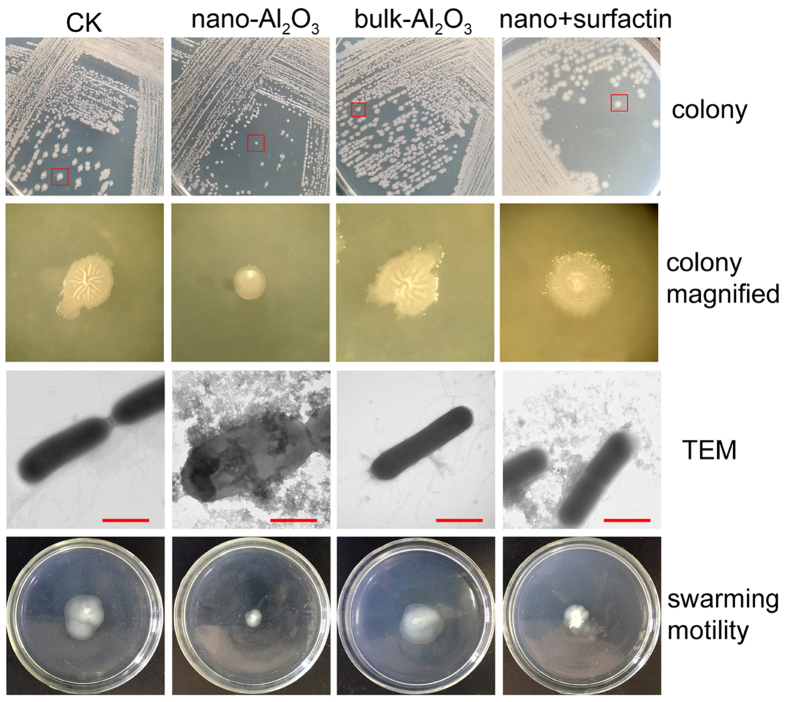
Influence of Al_2_O_3_ NP stress on the colony morphogenesis and motility of *B. subtilis* C01. CK indicates control treatment, nano-Al_2_O_3_ indicates 3 mM Al_2_O_3_ NPs were added in the 2216E agar plate or fermentation at 12 h, bulk- Al_2_O_3_ indicates 3 mM bulk-Al_2_O_3_ treatment, nano+surfactin indicates both 3 mM Al_2_O_3_ NPs and 20 μM commercial surfactin were added to the culture. Colony indicates the colony of C01 grew on a different plate, the red box indicates the colony selected for colony magnified analysis; TEM shows the cells with different treatment, red bar = 500 nm; swarming motility indicates growth of *B. subtilis* strains on media with 0.3% agar and different treatments. Bacteria were centrally inoculated onto the soft plates.

To evaluate the effect of Al_2_O_3_ NPs on *B. subtilis* C01 morphogenesis, C01 was grown on 2216E medium with or without 3 mM Al_2_O_3_ NPs. As shown in Fig. 3, C01 control populations on the agar medium develop colonies with robust morphology. However, the robustness of colony morphology was dramatically diminished on the 3 mM Al_2_O_3_ NP-containing agar medium. Colonies grown on the bulk-Al_2_O_3_-containing agar medium were similar to those on control agar medium (Fig. 3).

To investigate the effect of Al_2_O_3_ NPs on surface motility, bacteria were spotted onto the centers of 2216E soft agar swarm plates (0.3% of agar in the 2216E medium with or without 3 mM Al_2_O_3_ NPs). Within 6–8 h of incubation, the bacteria formed a colony of 2–4 cm diameter in control swarm plates (Fig. 3). However, the presence of 3 mM Al_2_O_3_ NPs significantly altered the motility of bacterial cells compared to control in which no Al_2_O_3_ NPs were present (Fig. 3). The results showed that Al_2_O_3_ NPs could influence the morphogenesis and motility due to the nano-size of this particle, as bulk-Al_2_O_3_ could not influence these phenotypes.

To determine whether Al_2_O_3_ NPs could affect the flagella, we carried out microscopic studies of cells collected from the control fermentation broth and broth treated by 3 mM Al_2_O_3_ NPs (Fig. 3). TEM results showed that cells from the control or bulk-Al_2_O_3-_added fermentation broth have a number of peritrichous flagella. However, under Al_2_O_3_ NPs treatment, Al_2_O_3_ NPs could attach to the membrane and cause the flagellar damage, because cells from such treatment showed flagella missing or agglomerate flagella, and floccules was found attached to the cells (Fig. 3). In addition, the cell morphology was significantly different in the presence and absence of Al_2_O_3_ NPs, and Al_2_O_3_ NPs could attach to the cell membrane (Fig. 3).

A consensus has emerged that swarming motility by *B. subtilis* requires or must be facilitated by the production of the lipopeptide surfactin^19^,^20^. Figure 3 shows that exogenously adding commercial surfactin in 2216E agar media could significantly restore the swarming motility and cell morphology compared to the Al_2_O_3_ NPs treatment. However, in the fermentation broth, surfactin did not reduce the flagellar damage, as the flagella of the cells in such treatment was also missing or agglomerate according to the TEM analysis (Fig. 3). Taken together, a relatively high surfactin production could restore and enhance swarming motility under Al_2_O_3_ NP stress, as exogenously adding commercial surfactin in Al_2_O_3_ NP treatment media could enhance the motility (Fig. 3).

### The mechanisms of toxicity for Al_2_O_3_ NPs on *B. subtilis*

To elucidate the mechanisms of toxicity for the Al_2_O_3_ NPs, a suite of assays measuring membrane potential, membrane damage, cellular ROS generation, and electron transport activity was employed in *B. subtilis* (see the Supporting Information for methods). The outcomes of these assays are shown in Fig. S3. Al_2_O_3_ NPs resulted in significant membrane damage and an increase in ROS generation at 3 mM and 10 mM concentrations (Fig. S3A,B), while bulk-Al_2_O_3_ did not result in membrane damage and ROS generation even by using 10 mM. This observation strongly agrees with the TEM analysis (Fig. 3) and growth inhibition results. Conversely, no disruption of membrane potential was observed in *B. subtilis* treated with either Al_2_O_3_ NPs or bulk-Al_2_O_3_ (Fig. S3C). Additionally, electron transport activity was not influenced by Al_2_O_3_ NPs and bulk-Al_2_O_3_ (Fig. S3D). These results highlight important mechanistic pathways of toxicity for Al_2_O_3_ NPs.

### Two-component signal systems and membrane proteins response to Al_2_O_3_ NP treatment

For genes involved in the response of Al_2_O_3_ NPs stress screening, transcriptome analysis was performed after 60 min when *B. subtilis* treated with 3 mM Al_2_O_3_ NPs, using control cells and bulk-Al_2_O_3_ as references (see Fig. S4 assay and Table S1 assay for the overview of cellular processes regulated at the transcriptional level).

**Figure 4:**
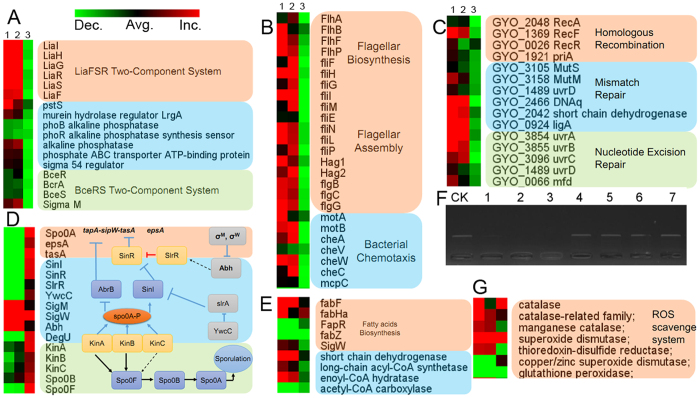
Gene expression profiles for several representative categories. Displayed are the relative expression levels of each gene. The numbers 1, 2, and 3 indicate Al_2_O_3_ NPs/control, Al_2_O_3_ NPs/bulk-Al_2_O_3_, and Al_2_O_3_/control, respectively. Color indications are red for increased expression, green for decreased expression and black for unchanged expression. (**A**) LiaRS TCS- and BceRS TCS-related genes; (**B**) Flagellar biosynthesis- and assembly-related genes; (**C**) DNA repair-related genes; (**D**) Biofilm formation-related genes; (**E**) Fatty acids biosynthesis and metabolism genes; (**F**) Using plasmid pET42a for DNA damage detection *in vitro*. CK indicates control, 1 indicates UV treatment, 2 indicates 10 mM Al_2_O_3_ NP treatment, 3 indicates 1 mM Al_2_O_3_ NP treatment, 4 indicates 0.1 mM Al_2_O_3_ NP treatment, 5 indicates 10 mM bulk-Al_2_O_3_ treatment, 3 indicates 1 mM bulk-Al_2_O_3_ treatment, 4 indicates 0.1 mM bulk-Al_2_O_3_ treatment; (**G**) ROS scavenger-related genes.

Two-component signal systems (TCS) are one means that bacteria have to respond to external stimuli. The transcriptome shows that the LiaRS TCS was involved in the adaptation to Al_2_O_3_ NPs stress, as most of the related genes were highly upregulated (Fig. 4A). The response regulator/histidine kinase pair LiaRS of *Bacillus subtilis*, together with its membrane-bound inhibitor protein LiaF, constitutes an envelope stress sensing module that is conserved in *Firmicutes* bacteria^21^. LiaRS strongly responds to the presence of a number of cell wall antibiotics, such as bacitracin^22^. The exact physiological role of *LiaI* and *LiaH* is not well understood, but the proteins seem to be involved in sensing and counteracting membrane damage^23^. Additionally, genes in BceRS TCS, which mainly regulated the cell envelope stress response^24^,^25^, were also upregulated in the adaptation to Al_2_O_3_ NP stress.

The membrane proteins are the integral part of the bacterial cell membrane to maintain the cell integrity. The transcriptome shows that many proteins integral to membrane were up-regulated, such as membrane protein OxaA, mechanosensitive channels. While inner membrane proteins involved in sporulation were down-regulated (Fig. S5). Taken together, these results indicate that membrane-related stress was one of the most important effects caused by Al_2_O_3_ NPs.

### Fatty acids biosynthesis and lipid metabolism gene response to Al_2_O_3_ NP stress

The transcriptome showed that the genes related to fatty acid biosynthesis (*FabF*, *FabHa*) were upregulated in response to the Al_2_O_3_ NPs stress (Fig. 4E), and *FapR*, involved in negative regulation of the fatty acid biosynthetic process^26^, was down-regulated (Fig. 4E). Increased abundance of the FabF elongation enzyme can increase the chain length of the resulting fatty acids^27^. Another key gene, extracytoplasmic function σ factor σ^W^, was also induced (Fig. 4E), which was reported have a function in regulation of FabHa and FabF^28^. Additionally, expressions of some genes involved in the lipid metabolism were influenced by Al_2_O_3_ NP stress (Fig. 4E). For example, long-chain acyl-CoA synthetase was induced, which was reported to activate fatty acids by thioesterification with coenzyme A. Fatty acyl-CoA molecules are then readily utilized for the biosynthesis of storage and membrane lipids^29^.

To test whether Al_2_O_3_ NPs could affect the fatty acids profiles, fatty acid profiles of cultures after exposure to Al_2_O_3_ NPs were analyzed and compared with the profiles of non-exposed cultures or cultures exposed to bulk-materials. Analysis revealed changes in membrane composition exclusively when cells were exposed to Al_2_O_3_ NPs at a concentration of 3 mM but not in cultures exposed to bulk-material (Table 1). The major changes were observed in proportions of *i*-C_13:0_, C_15:0_, *i*-C_14:0_-3OH, and C_18:0_ (Table 1).

### Expression of genes involved in flagellar assembly and chemotaxis enhanced in the presence of Al_2_O_3_ NPs

The transcription of flagellar biosynthesis genes (e.g., *FlhA*, *FlhB*, *FlhF,* and *flgBCG*) were highly upregulated, as well as most of the flagellar assembly genes (e.g., *FliE-I*) (Fig. 4B). Most prominent among the upregulated genes are the large *flgB* operon and the hag gene, both involved in motility. Flagella are constructed from over 20 different proteins that must be assembled with the correct order and in the correct stoichiometry^30^. To ensure proper assembly, flagellar gene expression is organized in at least two hierarchical levels defined here as “early-class” genes, recognized by σ^70^, and “late-class” genes, recognized by the alternative sigma factor σ^28 ^31. *FliE,* encoding the flagellar hook-basal body complex protein, is a classic Early-class flagellar genes, was upregulated at the point of 60 min (Fig. 4B). *MotA* and *MotB*, encoding the protein components of the stator of proton-driven motors, were also upregulated (Fig. 4B). Generally, FliM secretion liberates its cognate σ^28^ to direct expression of the late-class flagellar genes. However, *FliM* was also upregulated at this time (Fig. 4B). Considering that Al_2_O_3_ NPs could eliminate the flagella when flocculation occurred, the types of regulated genes indicated that the flagella damage caused by Al_2_O_3_ NPs occurred at different levels, and cells needed to express various genes belonging to different hierarchical levels to repair the flagellar system.

The transcriptomic data also show that many genes belonging to the chemotaxis system [e.g., chemoreceptor proteins (methylated chemotaxis proteins, MCPs), chemotaxis histidine kinase (CheA), and chemotaxis proteins (CheW and CheC)] are upregulated in the Al_2_O_3_ NP treatment (Fig. 4B). The response of the MCPs is transmitted via the CheA histidine kinase that is complexed, together with adaptor protein CheW, on the cytoplasmic side^32^. In addition, other genes that improve the cell motility and secretion were also upregulated such as *ClpX* (encoding ATP-dependent Clp protease proteolytic subunit) (Fig. 4B).

### Al_2_O_3_ NPs affect the biofilm- and sporulation-related genes

Biofilm formation is a social behavior that generates favorable conditions for sustained survival in the natural environment^33^. Generally, regulatory pathways that control biofilm formation include the Spo0A pathway, the SlrR–SinR epigenetic switch system [the YwcC and SlrA pathway and the Abh-extracytoplasmic function (ECF) RNA polymerase σ-factors pathway], and the DegS–DegU two-component system. The relationship of the four pathways is shown in Fig. 4D. The transcriptome analysis showed that most genes related to the Spo0A pathway (e.g., *KinA*-*C* and *spo0A*) were down-regulated, as well as *tapA*–*sipW*–*tasA* and *SinR* (Fig. 4D). Spo0A is a central transcriptional regulator that controls the expression of more than 100 genes, including those necessary for biofilm matrix gene expression and sporulation, by controlling the activity of the master regulator SinR, a repressor of the *eps* and *tapA*–*sipW*–*tasA* operons^34^.

Another pathway mediated by the TetR-type transcriptional repressor (YwcC) was down-regulated^35^. When YwcC receives an as-yet-unknown signal, slrA is derepressed and the matrix genes are induced by SlrA-mediated inactivation of SinR^35^. Meanwhile, the Abh- ECF σ factors pathway also showed a response to Al_2_O_3_ NP stress (Fig. 4D); the Abh protein regulates the transcription of *slrR*, further inactivates SinR and then induces the expression of matrix genes^36^. The transcription of *abh* is controlled by several extracytoplasmic function (ECF) RNA polymerase σ-factors, includingσ^M^, σ^W^ and σ^X ^36. ECF σ-factors are activated by a variety of external stimuli, including cell wall stress and specific antibiotics^37^.

At the same time, genes involved sporulation (e.g., *KinA-C*, *spo0F*, *spo0B*, and *spo0A*) were also suppressed in the Al_2_O_3_ NP treatment at 60 min. the percentage of sporulated cells was quantified, and the results showed that a high concentration of Al_2_O_3_ NPs could suppress the formation of spores within 72 h (Fig. S6).

### Upregulation of DNA damage repair-associated genes

Following Al_2_O_3_ NP treatment for 60 min, three group of genes (homologous recombination, mismatch repair, and nucleotide excision repair) belonging to the DNA damage repair system were upregulated (Fig. 4C). For example, RecA, which is central to genome integrity and is important for strand exchange during homologous recombination, stabilizing stalled replication forks, and induction of the SOS transcriptional response to DNA damage^38^, was upregulated in the treatment. Meanwhile, genes encoding DNA mismatch repair and nucleotide excision repair proteins were also upregulated (Fig. 4C).

A plasmid-based *in vitro* DNA damage assay^39^ was used to study the intrinsic potential of the Al_2_O_3_ NPs to damage double-stranded DNA. The gel electrophoresis results for various treatments are shown in Fig. 4F. The positive control used in this study was a UV-treated plasmid, which was completely degraded and appeared as smeared. Severe DNA damage was observed for Al_2_O_3_ NPs, which induced complete degradation of plasmid DNA (Fig. 4F). By contrast, bulk-Al_2_O_3_ had no effect on DNA damage, appearing similar to the negative control. This might be one way to induce the DNA repair response *in vivo*, because a previous study had demonstrated the attachment of Al_2_O_3_ NPs to the surface of the cell membrane and also their presence inside the cells due to formation of irregular-shaped pits and perforation on the surfaces of bacterial cells^40^. Furthermore, Al_2_O_3_ NPs also induced the ROS scavenge system response, as some of the catalase-related family genes and super oxide dismutase (SOD) were upregulated (Fig. 4G). The physiological analysis also showed that high concentrations of Al_2_O_3_ NPs could induce ROS in cells compared with bulk-Al_2_O_3_ treatment and controls (Fig. 4B) and also might result in DNA damage *in vivo*^9^. However, the exact mechanisms for DNA damage *in vivo* need further study.

### Systematic validation of transcriptome data using real-time PCR

To validate the transcriptome data and systematically analyze the expression of key genes during treatment with Al_2_O_3_ NPs and to further elucidate the mechanism of adaption of marine *Bacillus subtilis* C01 to Al_2_O_3_ NP stress, eighteen key genes were selected on the basis of their possible role in two-component signal systems, membrane integrality, stress response, flagellar assembly, motility, biofilm formation, DNA repair, transcriptional regulation, and surfactin biosynthesis for real-time PCR analysis (Fig. S7). The real-time PCR used the control treatment and bulk-Al_2_O_3_ treatment as references, separately. Meanwhile, we detected the gene transcripts at 60 min, 12 h, and 24 h after treatment with Al_2_O_3_ NPs. The quantitative gene expression results correlated with the trend of regulation observed in the transcriptome experiment. In general, the relative expression of genes in Al_2_O_3_ NPs/control were similar to those in Al_2_O_3_ NPs/bulk-Al_2_O_3_, and the genes showed different responses to Al_2_O_3_ NPs at different time points (Fig. S7). During the early stages of Al_2_O_3_ NP stress, *LiaR*, as a key gene in LiaRS two-component signal systems mainly regulating the cell envelope stress response, was highly upregulated. Prolonged Al_2_O_3_ NPs stress for 24 h leads to normal levels of *LiaR*. Genes involved in membrane integrality, SOD genes, and catalase genes were up-regulated last for a long time (last for 24 h). Genes involved in biosynthesis of flagellar components (*flgB* and *fliE*) exhibited a dramatic enhancement in their expression at 60 min after Al_2_O_3_ NP treatment; however, these genes recovered to the normal level of expression for continued exposure to Al_2_O_3_ NPs at 12 h and 24 h (Fig. S7). *RecA*, involved in DNA repair, was also highly induced in the early stage of Al_2_O_3_ NP stress (60 min); however, in the middle stage, *RecA* showed a dramatically decreased level of expression and then recovered to normal levels during the prolonged Al_2_O_3_ NP exposure for 24 h (Fig. S7).

Most of biofilm formation and sporulation-related genes (*DegS*, *SinR*, *KinA*, *Spo0A*, *tasA*, *epsA*, and *abrB*) were down-regulated in the early stages of Al_2_O_3_ NP stress; however, most of these genes were upregulated with prolonged with Al_2_O_3_ NP exposure at 12 h or 24 h. Interestingly, the expression of *SinR* showed sustained down-regulation. Among those genes, both SinR and AbrB could repress *tapA-sipW-tasA* and *epsA* expression. The prolonged stress leading to down-regulation of *SinR* might induce the expression of *tasA* and *epsA*, and the repression from AbrB to *tasA-epsA* might be inhibited by high expression of *spo0A* (Fig. S7), thereby inducing biofilm formation in shake cultures (Fig. 2A). However, the exact mechanisms need to be clarified in future studies.

More importantly, in this experiment, surfactin production was enhanced during the prolonged Al_2_O_3_ NP stress for 60 h (Fig. 1C). However, the transcriptome showed the genes involved in the surfactin biosynthesis (*srfAA-D*) showed no significant change in expression, as even the regulation gene (*comA*) was down-regulated in the early stages (data not shown). The real-time PCR results showed that *comA* and *srfA* were indeed down-regulated during the early stages of stress but were induced with prolonged stress, and both were upregulated at 24 h; however, *comA* also showed down-regulation at 12 h treatment (Fig. S7). These results were consistent with the phenotypes in the former experiments (Fig. 2B).

## Discussion

Recently, it has been widely accepted that NPs can offer a new strategy to tackle multidrug-resistant bacteria^41^,^42^. Many studies have focused on the antibacterial properties of NPs, and described the toxicity mechanisms of NPs against bacteria and drug-resistant bacteria^9^,^10^. However, the few existing reports on the defense mechanisms of tolerant bacteria against NPs are limited to *Mycobacterium smegmatis* with Cu-doped TiO_2_ NPs^43^, *B. subtilis* and *Pseudomonas putida* with nC_60_^44^, and *Cupriavidus metallidurans* CH34 with Al_2_O_3_ NPs^45^ and do not provide mechanistic insights by using full transcriptional analysis. Whether secondary metabolism biosynthesis could respond to NP stress and enhance the adaption of bacteria to NPs was unknown.

The Al_2_O_3_ NPs have been shown to attack the bacterial cell membrane, alter membrane permeability^15^,^46^, and even accumulate inside the bacterial cell^40^. In agreement with this report, in our study, Al_2_O_3_ NPs could attach to the cell membrane, affect the cell morphology and even cause membrane damage (Fig. 3). Meanwhile, toxicity analysis showed that relatively high concentrations of Al_2_O_3_ NPs could cause membrane damage compared with bulk-Al_2_O_3_. Further transcriptional analysis showed that at least 200 genes encoding proteins related to membrane components were regulated by Al_2_O_3_ NPs at the early stage of stress (Fig. S4), including genes involved in the LiaRS TCS^23^ and the BceRS TCS^24^,^25^, both of which were involved in sensing and counteracting membrane damage (Figs 4A and S7). Extracytoplasmic function (ECF) RNA polymerase σ-factors (σ^W^, σ^M^), involved in stress responses elicited by compounds that affect membrane integrity and/or fluidity, were also upregulated in the early stage of Al_2_O_3_ NP stress. Cells have evolved the ability to modify membrane lipid composition to acclimatize to membrane stress^28^. In this sense, Kingston *et al*.^28^ suggested that the σ^W^-dependent stress response in *B. subtilis* could regulate the fatty acids biosynthesis and reduce the membrane fluidity. Accordingly, our results show that the genes involved in fatty acids biosynthesis and the fatty acid profiles were changed with Al_2_O_3_ NP stress, and *i*-C_13:0_, C_15:0_, *i*-C_14:0_-3OH, and C_18:0_ were induced, which contribute to antioxidant stress and improve the stability of the membrane^28^,^47^, however, the results were different to nC_60_ treatment, under which *B. subtilis* showed an increase in membrane fluidity^44^. In this study, ROS generation was also induced in the early stage, which might be related to the membrane damage. In other bacteria, Al_2_O_3_ NP was also found to cause the membrane damage and induce the intracellular ROS in *E. coli* MG1655 and *Cupriavidus metallidurans* CH34^45^. Taken together, these results suggest that membrane-related stress was one of the most important effects caused by Al_2_O_3_ NPs.

It was reported that some NPs such as Cu NPs^9^ and Ag NPs^48^ could damage DNA and cause growth inhibition of bacteria. However, whether Al_2_O_3_ NPs have such properties remains unclear. In this study, the transcriptional analysis showed DNA damage repair-associated genes were highly upregulated in the early stage of Al_2_O_3_ NP stress. Additionally, we found that a high concentration of Al_2_O_3_ NPs could damage DNA *in vitro* (Fig. 4F). Furthermore, NPs generated ROS could cause DNA damage and induce DNA repair genes^9^. Coupled with the membrane damage and the induction of cellular ROS, Al_2_O_3_ NPs probably induce the DNA damage *in vivo*. This result is in accordance with previously published data, which Cu NPs induce DNA damage in *E. coli*^9^, however, the exact mechanism for DNA damage needs further study.

Flagella enable *Bacillus subtilis* to move towards favorable environments or avoid harmful stimuli during swimming^20^. To avoid the stress of Al_2_O_3_ NPs, *B. subtilis* had to regulate the expression of flagellar biosynthesis genes as well as most of the flagellar assembly genes (Fig. 4B). In this study, TEM analysis showed that Al_2_O_3_ NPs could attach to the membrane and flagella, causing flagellar damage. As a result, *B. subtilis* upregulates the expression of the flagellar biosynthesis genes and flagellar assembly genes to repair the impaired flagella. Although the flagellar-related genes showed high expression following Al_2_O_3_ NP treatment, the motility was not enhanced. This is probably because flagellar damage restricted the motility or the attachment of Al_2_O_3_ NPs to cells increased the resistance to motility.

Swarming and sliding motility by *B. subtilis* were shown to require or be facilitated by the production of the lipopeptide surfactin^20^. In agreement with their report, our data showed that surfactin could restore and enhance swarming motility under Al_2_O_3_ NP stress (Fig. 3). During Al_2_O_3_ NP-treated fermentation, the induced surfactin production may alleviate the motility restriction to enhance chemotaxis in an attempt to eliminate the Al_2_O_3_ NPs, as chemotaxis genes were also induced in this experiment. Interestingly, the surfactin produced could also reduce the surface tension^49^, which may help to wash Al_2_O_3_ NPs away from cells. However, this needs to be determined in further studies.

Bacterial biofilms are multicellular communities in which cells are held together by an extracellular matrix to strengthen the adaption to various environmental factors^50^,^51^. In full agreement with these reports, our results showed a series of genes involved in biofilm formation were induced in different stages of Al_2_O_3_ NP treatment. In the early stage, the YwcC–SlrA pathway and the Abh-ECF σ factors pathway were induced for a quick response to Al_2_O_3_ NPs stress, while in the late stage, the spo0A pathway was activated and up-regulated *tasA* and *epsA* genes (Figs 4D and S7). Similar results were found in *Shewanella oneidensis* MR-1 response to Cu-doped TiO_2_ NPs. *S. oneidensis* MR-1 could produce a large amount of extracellular polymeric substances (EPS) under NP stress, especially extracellular protein^43^. Surfactin, produced by constituent cells of the biofilm, was the first molecule identified as an inducer of matrix gene expression^52^. A recent study showed that surfactin could trigger the biofilm formation of *B. subtilis* in melon phylloplane^51^. The induction of surfactin in the late stage of Al_2_O_3_ NP stress might contribute to the biofilm formation to alleviate Al_2_O_3_ NP stress by separating *B. subtilis* from the Al_2_O_3_ NPs (Fig. S8).

In summary, our transcriptome and physiological analyses suggest that the attachment of Al_2_O_3_ NPs to the membrane of *B. subtilis* along with flagellar damage would initiate sensing and counteracting membrane damage. These responses also lead to changes in the membrane fatty acids profile, induction of DNA repair and the ROS scavenger system, and enhancement of flagellar biosynthesis to repair the organism, resulting in optimal conditions for *B. subtilis* growth and adaptation to Al_2_O_3_ NPs in the early stage of stress. Furthermore, biofilm formation and surfactin biosynthesis were induced in the late stage to adapt to or avoid the stress (Fig. S8).

## Methods

### Strains and culture conditions

Marine *Bacillus sp.* strain C01, isolated from Weihai, was grown at 30 °C in Landy medium^16^. A 1% inoculum volume of culture was used to inoculate a 250 mL flask containing 100 ml of Landy medium, which was incubated for 72 h on an orbital shaker (150 rpm) at 30 °C. Al_2_O_3_ NPs were added as an inductor at different times.

### Preparation of Al_2_O_3_ NPs and bulk-Al_2_O_3_ suspensions

Al_2_O_3_ NPs and bulk-Al_2_O_3_ were purchased from Shenzhen Crystal Material Chemical Co., Ltd (Shenzhen, China). The suspensions of Al_2_O_3_ NPs and bulk-Al_2_O_3_ were diluted with ultra-pure water. To avoid aggregation, the suspensions were ultra-sonicated for 15 min in sealed sterile tubes before addition to the cell culture. The following concentrations were used in the experiment: 0, 0.3, 1, 3, and 10 mmol/L. The size of Al_2_O_3_ NPs were detected by using Scanning Electron Microscope (Nova NanoSEM 450)^15^.

### Isolation of surfactin

Crude surfactin was isolated by adding concentrated hydrochloric acid to the Landy media after removing the biomass by centrifugation. A precipitate was formed at pH 2 which could be collected, dried, and extracted with dichloromethane. The solvent was removed under reduced pressure to give an off-white solid. Further purification was achieved by recrystallization. The dichloromethane extract was dissolved in distilled water containing sufficient NaOH to produce a pH of 8. This solution was filtered and titrated to pH 2 with concentrated HCl. The white solid was collected as a pellet after centrifugation.

### Quantitative analysis of surfactin by HPLC

The isolated surfactin was dissolved in 1 mL of methanol followed by charcoal treatment and passed through a 0.22- μm-pore filter. The filtrate was subjected to HPLC on a reversed-phase column (RP-C18, 5 μm, 4*250 mm; Merck). The column was eluted at a flow rate of 1.0 mL/min with acetonitrile-water (80:20, v/v) and monitored at 214 nm. The concentration of surfactin was determined with a calibration curve made with authentic surfactin purchased from Sigma (S3523).

### Quantitative analysis of biofilm formation

At specific times, planktonic cells were removed, biofilm cells were stained with 2 ml of 0.3% crystal violet for 10 min, washed with distilled water, and air dried. The crystal violet in the biofilm cells was solubilized with 2 ml of 70% ethanol, and the optical density at 570 nm (OD_570_) was measured^53^.

### Cell morphology and motility study

The effect of Al_2_O_3_ NPs on *Bacillus* cell morphology and the attachment of Al_2_O_3_ NPs to *B. subtilis* were studies using transmission electron microscopy (TEM)^15^.

Bacterial motility over a surface was analyzed by spotting 2 μl culture of each strain grown overnight (~10^5^ cells) onto the center of soft agar plates (2216E media with 0.3% agar) with different treatments. Plates were incubated at 30 °C in a humidified chamber for 6–8 h.

## Additional Information

**How to cite this article**: Mu, D. *et al*. Physiological and transcriptomic analyses reveal mechanistic insight into the adaption of marine *Bacillus subtilis* C01 to alumina nanoparticles. *Sci. Rep.* **6**, 29953; doi: 10.1038/srep29953 (2016).

## Supplementary Material

Supplementary Information

## Figures and Tables

**Table 1:** The effect of Al_2_O_3_ NPs on membrane composition.

**Peak Name**^a^	**CK**	**bulk-Al**_**2**_**O**_**3**_	**Al**_**2**_**O**_**3**_ **NPs**
%12:0 ISO	ND	0.92 ± 0.03	0.65 ± 0.03
%13:0 ISO	ND	ND	0.16 ± 0.02**
%13:0 ANTEISO	ND	0.68 ± 0.04	0.48 ± 0.11
%14:0 ISO	10.77 ± 0.15	11.93 ± 0.39	9.53 ± 0.27
%14:0	ND	0.85 ± 0.07	0.82 ± 0.04
%15:0 ISO	9.62 ± 0.08	9.43 ± 0.11	9.67 ± 0.09
%15:0 ANTEISO	34.86 ± 0.41	35.76 ± 0.98	34.26 ± 0.83
%15:0	ND	ND	0.29 ± 0.04**
%14:0 ISO 3OH	ND	ND	0.3 ± 0.07**
%16:0 ISO	20.41 ± 0.28	19.52 ± 0.74	19.5 ± 0.66
%16:0	8.55 ± 0.13	7.11 ± 0.11	7.94 ± 0.51
%17:0 ISO	6.9 ± 0.09	5.18 ± 0.17	6.32 ± 0.74
%17:0 ANTEISO	8.89 ± 0.11	6.96 ± 0.13	8.27 ± 0.28
%18:0 ISO	ND	0.54 ± 0.05	0.66 ± 0.06
%18:0	ND	0.53 ± 0.03	1.14 ± 0.09*

Data are presented as the average of three trials (±standard error, *P ≤ 0.05, **P).

^a^Data derived from FAME analysis of the following treatment: CK indicates control, Al_2_O_3_ NPs indicates 3 mM Al_2_O_3_ NPs treated for 24 h, and bulk-Al_2_O_3_ indicates 3 mM bulk-Al_2_O_3_ treated for 24 h.
