# Supplementary material for: Physiological and transcriptomic analyses reveal mechanistic insight into the adaption of marine Bacillus subtilis C01 to alumina nanoparticles
Source: Sci Rep. 2016 Jul 21;6:29953. doi: 10.1038/srep29953 (PMC4954987; doi:10.1038/srep29953)
Supplement: Supplementary Information [file srep29953-s1.pdf]

Physiological and transcriptomic analyses reveal a mechanistic insight into the adaption of marine *Bacillus subtilis* C01 to alumina nanoparticles

Dashuai Mu[1,2], Xiuxia Yu[2], Zhenxing Xu[2], Zongjun Du[1,2*], Guanjun Chen[1,2*]

[1] State Key Laboratory of Microbial Technology, Shandong University, Jinan 250100, PR China

[2] College of Marine Science, Shandong University (Weihai), Weihai 264209, PR China

*Address correspondence to:

Guanjun Chen, E-mail: guanjun@sdu.edu.cn; Tel. (+86) 06315688199; Mailing address: State Key Laboratory of Microbial Technology, Shandong University, 27 Shandanan Road, Jinan, Box 250100, PR China.

Zongjun Du, E-mail: duzongjun@sdu.edu.cn; Tel. (+86) 06315688303. Mailing address: College of Marine Science, Shandong University (Weihai), 180 Wenhuaxi Road, Weihai, Box 264209, PR China

Supplementary Table S1

Table S1. Realtime PCR primers

primer name	sequences
LiaR-real-f	GAAGGTGTTCGGCTTGCTG
LiaR-real-r	CTCGCCTATTTCTTTGTTTGT
flgB-real-f	TGCGAGCGGAGATACATCG
flgB-real-r	TACCGTTCATTCTTTCAACCA
fliE-real-f	ATTTCTCCTTTTCAGGTTC
fliE-real-r	AGCTACTTGGGACTCATTT
swrA-real-f	TATTCAGCAGGTATTTGGT
swrA-real-r	ATTCCTAGTCGCTTATGGT
RacA-real-F	CAAGTGAACTCGGCGTCTC
RacA-real-R	TTCGTCCTGCCTTTGCTG
DegS-real-F	CAAATGCTGGCAAATGTTA
DegS-real-R	AAGCCGCTGATCCTCTGTT
KinA-real-f	TATCTGCCAACTCCAAACT
KinA-real-r	CAACCGTTCATAATCATCC
Spo0A-real-F	TTGATTCCATTTCCTCGTT
Spo0A-real-r	AAGCCTTATGCTCTAACCTCA
AbrB-real-f	ACTGCGTCGTACTCTTGGA
AbrB-real-r	TTTCGCTGATGATTTGCTC
srfa-real-f	TACAAGGGATACAAGCAGAA
srfa-real-r	AAGGCAGTCGCATAGTCAA
sinR-f	GGGGTAGCGAAGTCTTATT

sinR-R	CCATCGTATTCGGTTTCAT
tasA-F	AAGATTTCCTCAGCCAGTT
tasA-r	GATTGTCAAGCCGTTCCAC
epsA-F	CATTTCACCGACCTACCAG
epsA-R	CCGCCTTAACTTCTTCCAT
MetK-real-f	AAATTGAAGCGATTGGTGC
MetK-real-r	CAGGGTGATGCTGAGTTGA
oxaA-f	TAGCGAAATTGACGGGAGA
oxaA-r	CCAACGGATTGACACCATG
YnaI-f	TTGAAAAGGTAACTGGGTT
YnaI-r	CCTCAATGTAAGAAATGCC
KatA-f	CGTGCCTGGTATTGATGTT
KatA-r	GTAGAAGTGCTCGATTTGG
sodA-f	CAAGATTCTCCGCTTTCAG
sodA-r	GTCCCAGTTTACAACATTCCA

Supplementary Table S2

Table S2. Part of the genes most prominently induced or repressed by Al_2O_3 NPs

Regulation	P-value	Product	Component	GO Process
Up	2.75E-07	SsuA protein;	plasma membrane	sulfur compound metabolic process
Up	8.12E-05	cephalosporin C deacetylase;	-	carbohydrate metabolic process
Up	1.83E-06	hypothetical protein;	-	signal transduction
Up	2.30E-05	acetoin cleaving system dihydrolipoyllysine-residue acetyltransferase;	-	acetoin catabolic process
Up	0	peptidase propeptide and ypeb domain-containing protein;	-	-
Up	0	protein LiaI;	membrane	-
Up	0	protein LiaH;	-	-
Up	4.35E-44	hypothetical protein;	plasma membrane	transport
Up	0	glutamate synthase [NADPH] small chain;	-	L-glutamate biosynthetic process
Up	0	protein LiaG;	-	response to stress
Up	0	sensory transduction protein LiaR;	cytoplasm	two-component signal transduction system (phosphorelay)
Up	0	sensor protein LiaS;	plasma membrane	Signal transduction by phosphorylation
Up	0	protein IolH;	-	-
Up	6.96E-121	hypothetical protein;	-	transport
Up	0	glutamate synthase [NADPH] large chain;	-	glutamine metabolic process
Up	1.16E-224	ribose transport ATP-binding protein RbsA;	plasma membrane	ATP catabolic process
Up	0	fructose-1,6-bisphosphate aldolase;	-	fructose 1,6-bisphosphate metabolic process
Up	4.05E-62	PstS protein;	plasma membrane	phosphate ion transport
Up	3.90E-29	protein IolI;	-	-

	0			
Up	0	glucosamine-6-phosphate isomerase;	-	N-acetylneuraminate catabolic process
Up	0	protein LiaF;	plasma membrane	-
Up	1.19E-242	inositol 2-dehydrogenase;	-	inositol catabolic process
Up	1.87E-150	PTS system beta-glucoside-specific transporter subunit IIBCA;	plasma membrane	phosphorylation
Up	3.88E-184	D-ribose-binding protein;	plasma membrane	carbohydrate transport
Up	2.47E-74	orotidine 5'-phosphate decarboxylase;	-	de novo' UMP biosynthetic processss
Up	0	hypothetical protein;	plasma membrane	regulation of transcription, DNA-dependent
Up	0	hypothetical protein;	integral to membrane	regulation of transcription, DNA-dependent
Up	0	UDP-N-acetylenolpyruvoylglucosamine reductase;	cytoplasm	oxidation-reduction process
Up	0	stress response protein NhaX;	-	response to stress
Down	5.04E-06	hypothetical protein;	integral to membrane	sporulation resulting in formation of a cellular spore
Down	2.56E-09	stage III sporulation protein AD;	integral to membrane	sporulation resulting in formation of a cellular spore
Down	2.71E-11	hypothetical protein;	plasma membrane	-
Down	0.000223336	spore coat protein YuzC;	viral capsid	-
Down	4.90E-05	3-oxoadipate CoA-transferase subunit B;	-	metabolic process
Down	0.000476758	acetate CoA-transferase subunit alpha;	-	metabolic process
Down	0	histidyl-tRNA synthetase 2;	cytoplas	histidine biosynthetic process

			m	
Down	3.86E-201	ATP phosphoribosyltransferase;	cytoplasm	histidine biosynthetic process
Down	4.47E-176	histidinol dehydrogenase;	-	histidine biosynthetic process
Down	1.38E-100	lipase;	extracellular region	lipid catabolic process
Down	8.44E-108	stage II sporulation protein P;	-	sporulation resulting in formation of a cellular spore
Down	3.44E-156	sigma-E processing peptidase SpoIIGA;	plasma membrane	sporulation resulting in formation of a cellular spore
Down	0	sacPA operon antiterminator;	-	positive regulation of transcription, DNA-dependent
Down	1.72E-14	germination and sporulation membrane protein YkvU;	integral to membrane	sporulation resulting in formation of a cellular spore
Down	0	stage II sporulation protein Q;	integral to membrane	sporulation resulting in formation of a cellular spore
Down	4.06E-255	sporulation protein YhfM;	-	-
Down	2.51E-110	stage II sporulation protein B;	integral to membrane	sporulation resulting in formation of a cellular spore
Down	0	hypothetical protein;	outer membrane	peptidoglycan turnover
Down	9.59E-47	hypothetical protein;	integral to membrane	sporulation resulting in formation of a cellular spore
Down	1.16E-122	forespore-specific transcriptional regulator RsfA;		sporulation resulting in formation of a cellular spore
Down	1.30E-11	spore coat protein CotJB;	viral capsid	-
Down	1.47E-84	RNA polymerase sigma-E factor;	-	regulation of transcription, DNA-dependent;sporulation resulting in formation of a cellular spore

Down	1.84E-31	sporulation integral membrane protein YlbJ;	integral to membrane	sporulation resulting in formation of a cellular spore
Down	0	L-lactate permease;	integral to plasma membrane	lactate transmembrane transport
Down	0	N-acetyl-gamma-glutamyl-phosphate reductase;	cytoplasm	arginine biosynthetic process;GO:0055114//oxidation-reduction process
Down	3.23E-37	sporulation-specific polysaccharide deacetylase PdaB;		sporulation resulting in formation of a cellular spore;GO:0045493//xylan catabolic process
Down	0	subtilisin;	extracellular region	fibrinolysis
Down	0	L-lactate dehydrogenase;	cytoplasm	cellular carbohydrate metabolic process
Down	4.44E-95	stage III sporulation protein AH;	integral to membrane	sporulation resulting in formation of a cellular spore
Down	0	argininosuccinate synthase;	cytoplasm	arginine biosynthetic process
Down	6.26E-85	membrane protein;	-	-
Down	0	nitrite transporter;	plasma membrane	transport
Down	8.15E-17	sporulation integral membrane protein YtvI;	plasma membrane	-

Supplementary Figure S1

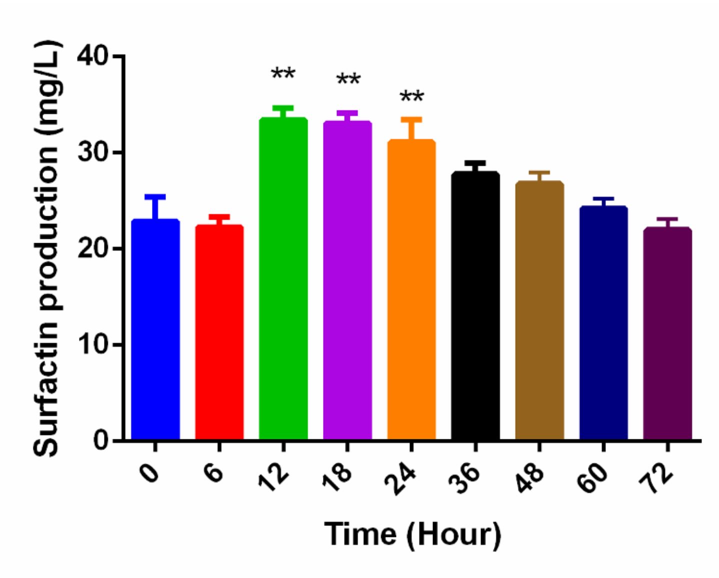

Figure S1. Surfactin production of different induction times (** P<0.01, n=3).

Supplementary Figure S2

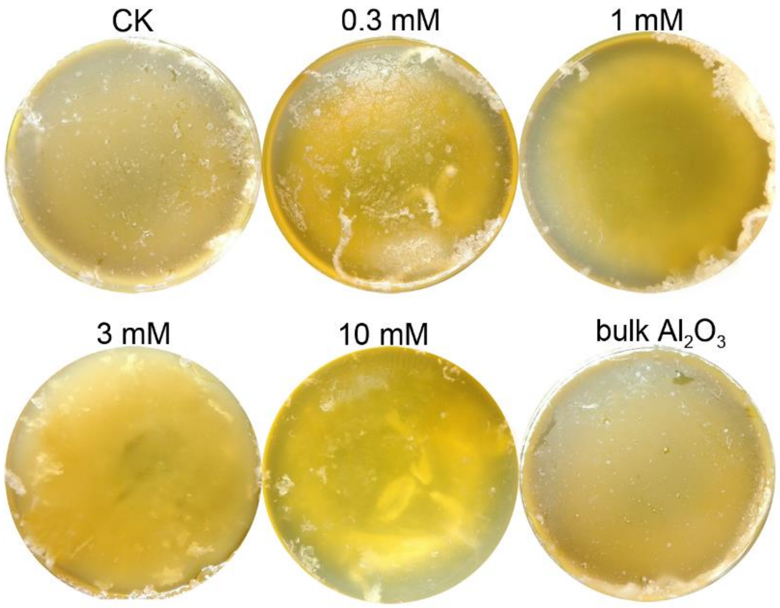

Figure S2. Phenotypic analysis of biofilm formation on the surface of broth. The fermentation broth was 100 ml in the 250 ml flask and was treated with different concentrations of Al_2O_3 NPs for stationary culturing for 12 h. CK indicates control, 0.3 mM, 1 mM, 3 mM, 10 mM indicate various concentrations of Al_2O_3 NPs, bulk-Al_2O_3 indicates 10 mM bulk-Al_2O_3.

Supplementary Figure S3

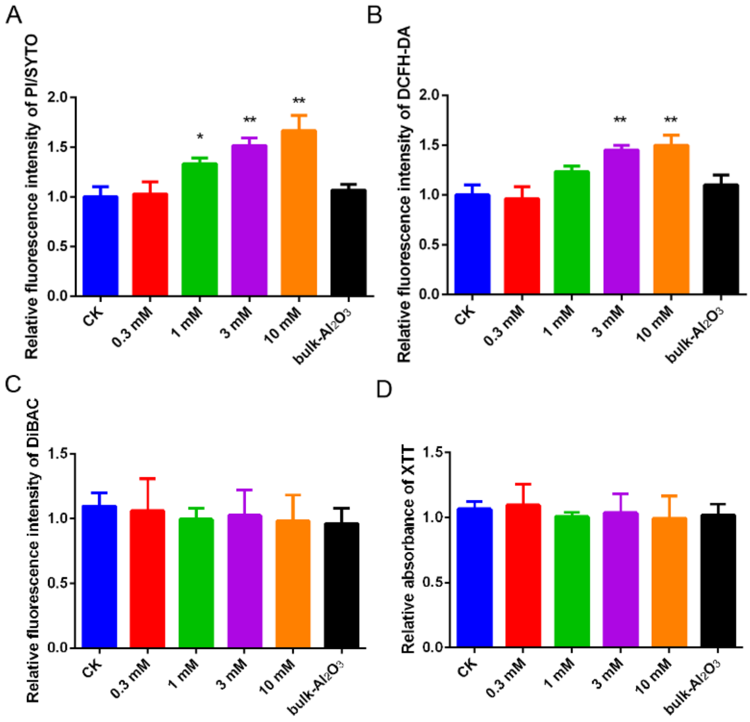

Figure S3. A suite of toxicity assays was used to elucidate the mechanisms of toxicity of Al_2O_3 NPs. Cells were treated with Al_2O_3 NPs ranging from 0.3 to 10 mM for 24 h before being treated with PI/SYTO and DCHF-DA to assess membrane damage and biotic ROS generation. CK indicates the control treatment with PBS alone (* $P<0.05$, ** $P<0.01$, n=3)

Supplementary Figure S4

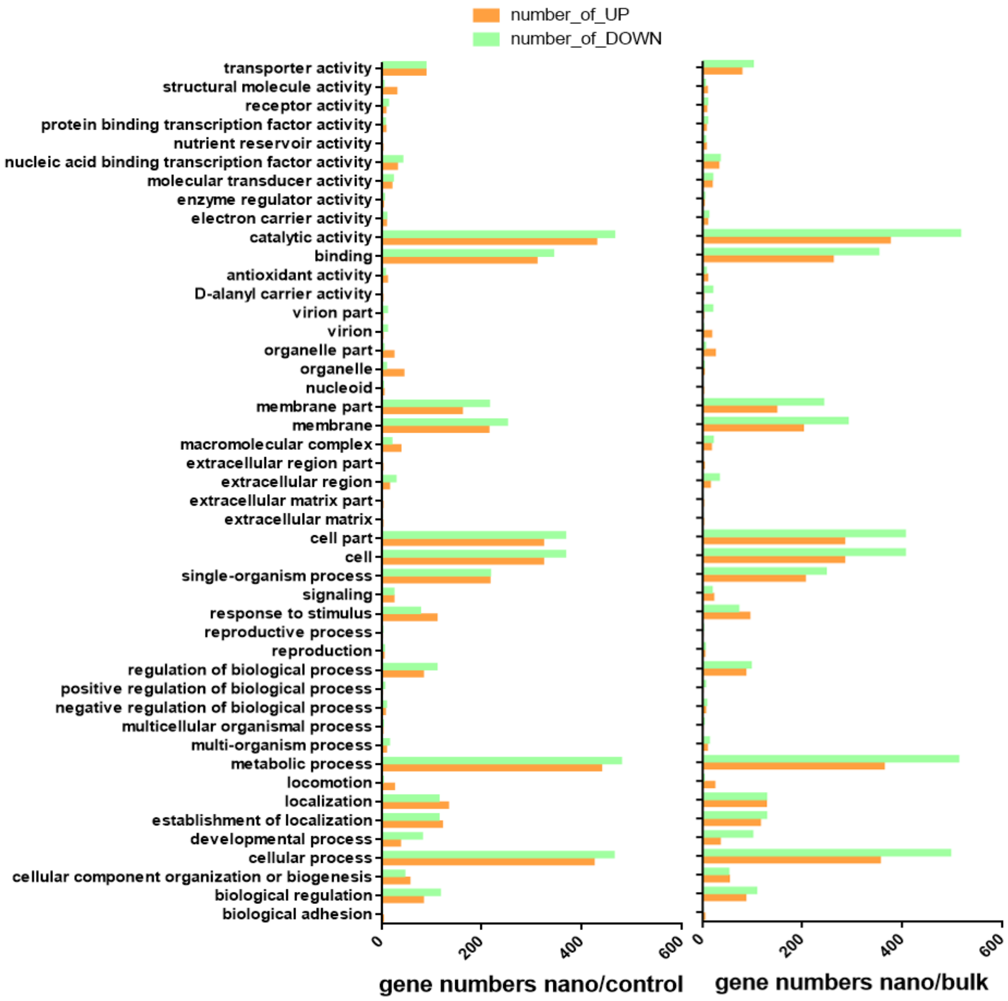

Figure S4. GO clustering analysis of differentially regulated genes following Al_2O_3 NP treatment. Number of regulated genes that are differentially expressed after different treatments. Gene numbers nano/control indicates gene expression under Al_2O_3 NP treatment compared with those under control. Gene numbers nano/bulk indicates gene expression under Al_2O_3 NPs treatment compared with those under bulk-Al_2O_3 treatment. The orange bars represent the number of upregulated genes and the green bars represent the number of down-regulated genes. The left bars represent the control used as a reference, and the right bars indicate bulk-Al_2O_3 used as a reference.

Supplementary Figure S5

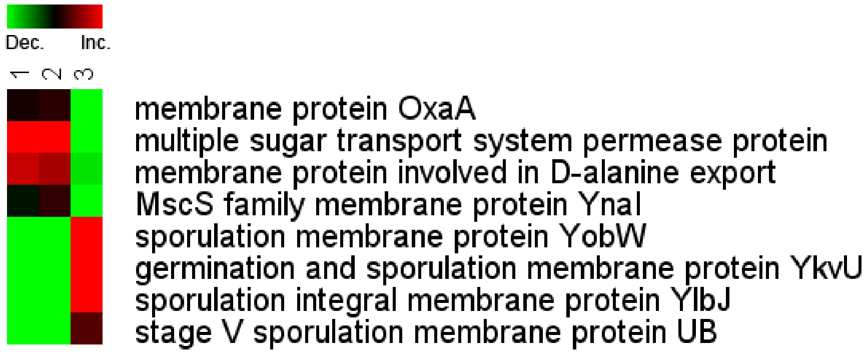

Figure S5. Gene expression profiles for membrane proteins. Displayed are the relative expression levels of each gene. The numbers 1, 2, and 3 indicate Al_2O_3 NPs/control, Al_2O_3 NPs /bulk-Al_2O_3, and Al_2O_3/control, respectively. Color indications are red for increased expression, green for decreased expression and black for unchanged expression.

Supplementary Figure S6

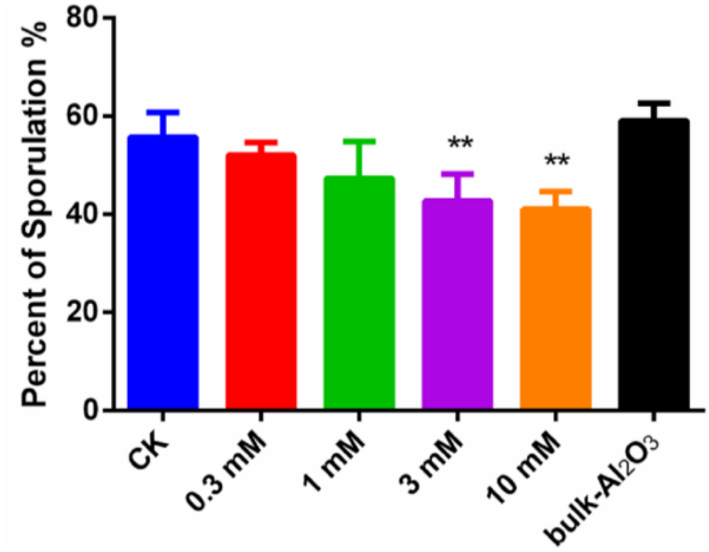

Figure S6. Quantification of sporulation under various treatment. Cells were treat with Al_2O_3 NPs ranging from 0.3 to 10 mM for 60 h before sporulation analysis, CK means control, and bulk-Al_2O_3 means 10 mM bulk-Al_2O_3 (** $p<0.01$, n=3).

Supplementary Figure S7

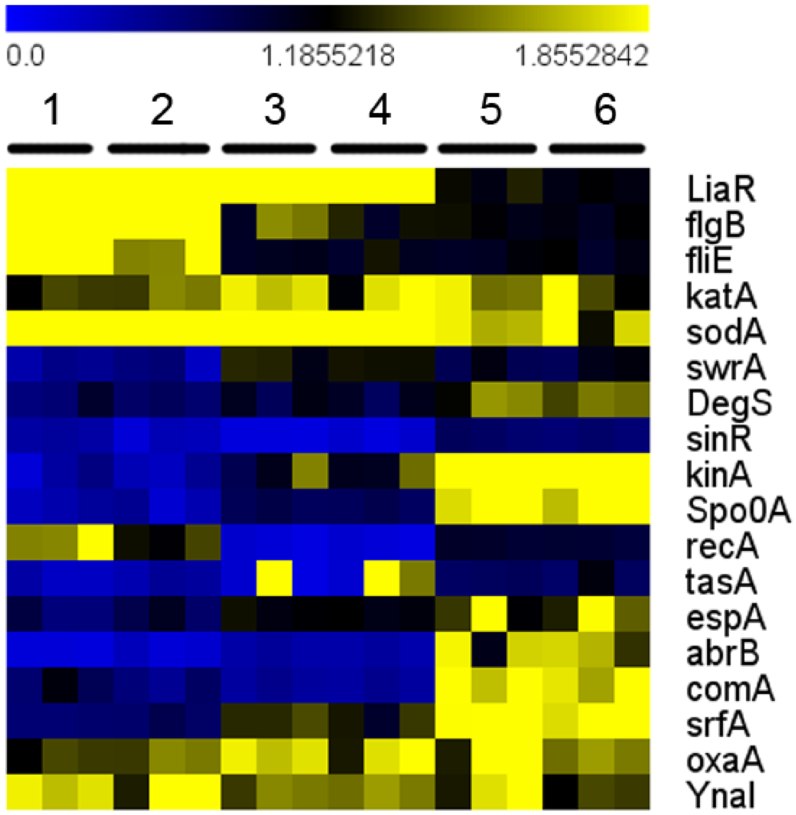

Figure S7. Transcription analysis of the response of key genes to 3 mM Al_2O_3 NPs at different times. All the 14 selected genes were detected using real-time PCR and presented as a heat map using GENESPRINGGX 7.3.1 software (Agilent Technologies). The number 1 indicates the relative expression of genes in the Al_2O_3 NP treatment compared to control at 60 min, number 2 indicates Al_2O_3 NPs/bulk-Al_2O_3 at 60 min; number 3 and 4 indicate Al_2O_3 NPs/control and Al_2O_3 NPs/bulk-Al_2O_3 at 12 h, respectively; Number 5 and 6 indicate Al_2O_3 NPs/control and Al_2O_3 NPs/bulk-Al_2O_3 at 24 h, respectively. The relative expression is shown as a mean value, from 0.0 to 1.8552842, in blue to yellow (n=3).

Supplementary Figure S8

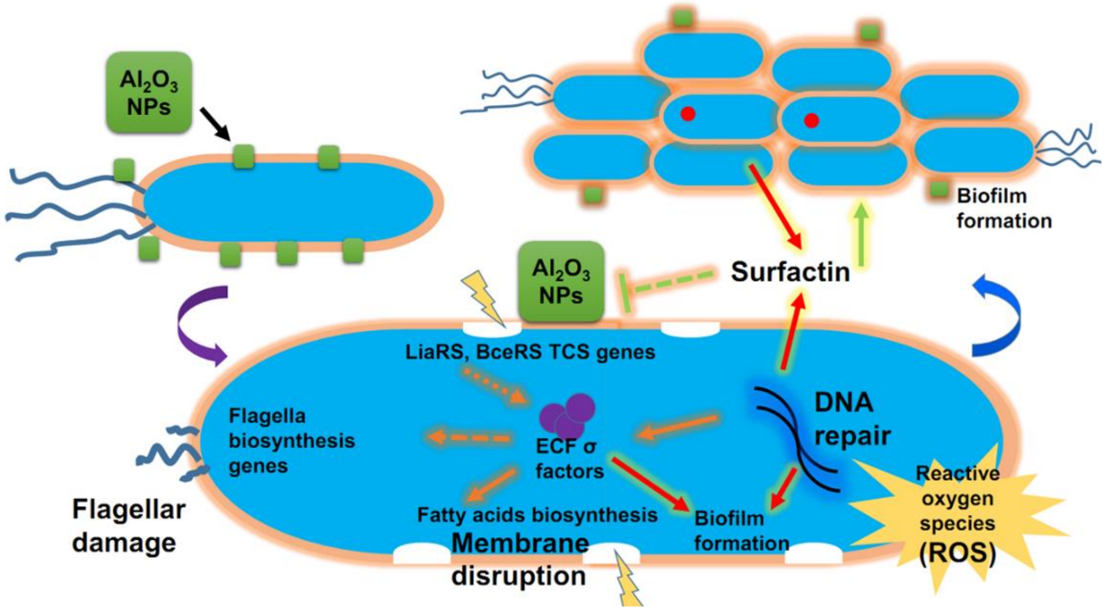

Figure S8. Schematic representation of the adaption of *B. subtilis* to Al_2O_3 NP stress. Al_2O_3 NPs (green square) attached to the membrane and flagella of *B. subtilis* (blue rod with orange cell wall), causing membrane and flagella damage along with the generation of ROS and DNA damage. *B. subtilis* responds to such stress through the TCS (e.g., LiaRS and BceRS) and DNA repair system and invokes the flagella biosynthesis and assembly system and fatty acids biosynthesis to repair the impaired cells through ECF σ factors (purple cycles). To adapt to such stress or avoid the stress, *B. subtilis* further activates the biofilm formation system and surfactin biosynthesis, showing an induction of surfactin production and biofilm formation. The solid lines indicate data supported by our own experiments and other reports, the dotted lines indicate hypothetical steps.

Supplementary Results

Transcriptome analysis of the Al_2O_3 NP treatment and overview of cellular processes regulated at the transcriptional level

To assess genes involved in the response to Al_2O_3 NPs, transcriptome analysis was performed after 60 min of treatment with 3 mM Al_2O_3 NPs, using control cells and bulk-Al_2O_3 as references (Fig. S4). Approximately 44% of the genes were found to be differentially expressed after treatment with Al_2O_3 NPs for 60 min. A total of 722 genes exhibited an increased relative messenger RNA (mRNA) level, whereas 876 genes exhibited a decreased relative mRNA level (Fig. S4).

The genes most prominently induced by Al_2O_3 NPs are involved in signal transduction, the two-component signal transduction system, response to stress, and carbohydrate metabolism (Table S2). The most strongly repressed genes are involved in sporulation, the histidine biosynthetic process, and lipid catabolism (Table S2). Analysis of the transcriptome data on overrepresented functional categories in clusters of up- and down-regulated genes revealed that membrane proteins, catalytic activity, metabolic processes, and cellular processes were enriched mostly among upregulated genes but also among some down-regulated genes (Fig. S4). Signal transduction, stress response, and motility genes were prominent categories among the upregulated genes, which also encompassed many genes encoding enzymes that are involved in flagellum assembly, DNA repair, amino acids, fatty acids and cell wall components. Surfactin biosynthesis genes were not significantly upregulated at this time point. Some genes involved in sporulation were down-regulated.

Supplementary Methods

Suite of toxicity assays

Membrane damage assay. To study the magnitude of bacterial membrane damage, a live/dead BacLight Bacteria Viability kit (L7012, Molecular Probe) was utilized. PI and SYTO 9 were mixed according to the manufacturer's instructions [1]; 0.15 µl of 3.34 mM SYTO 9 dye and 0.15 µl of 20 mM propidium iodide were added to 100 µl of a log-phase cell culturel that had been treated with Al_2O_3 NPs for 24 hours in a 96-well plate. In this experiment, PI (red fluorescence signal) specifically penetrates cells with compromised membrane whereas SYTO 9 (green fluorescence signal) non-selectively stains the DNA of every cell. The fluorescence signal was measured using a microplate reader (SpectraMax MS, Molecular Devices, CA) with excitation at 485 nm and the emission at 530 nm (SYTO 9) and 630 nm (PI). The ratio of the green/red fluorescence signals was used to determine the level of membrane damage, where a larger green/red value indicates less cell membrane damage.

Cellular ROS generation. To study cellular ROS generation, a stock solution of 2′,7′-dichlorodihydrofluorescein diacetate (DCFH-DA, Molecular Probes, D399) was dissolved in ethanol to yield a concentration of 1.5 mg/mL. For biotic ROS detection, DCFH-DA crosses cell membrane and is hydrolyzed by intracellular esterases to nonfluorescent dichlorofluorescin (DCFH). DCFH is then converted to the highly fluorescent 2′,7′-dichlorofluorescein (DCF) in the presence of intracellular reactive oxygen species. In this experiment, a log-phase culture (OD600=0.5) of *B. subtilis* was treated with a gradient of concentrations of Al_2O_3 NPs (0-10 mM) for 24

hours before being mixed with DCFH-DA (final concentration of 45 ng/mL). Three replicates were performed at each concentration. One hundred microliters of each mixture were then aliquoted into 96 well-plates and the fluorescent signal was measured using a microplate reader (excitation/emission at 485/530 nm; SpectraMax MS, Molecular Devices, CA).

Membrane potential assay and Electron transport activity assay were using the DiBAC method and XTT method, separately [1].

In vitro DNA damage assays

Plasmid pET42a was incubated with a gradient of concentrations of Al_2O_3 NPs for 24 h in purified water. Centrifugation at 15,000 rpm for 30 min was used to separate plasmid DNA from residual Al_2O_3 NPs. The supernatant containing the plasmid DNA was collected and loaded into a 1.2% Tris-acetate EDTA buffer (TAE) agarose gel. To induce random nicking and DNA fragmentation (positive control), the plasmid was treated with a xenon arc UV-B lamp for 10 min. For the negative control, pET42a was loaded onto the electrophoresis gel.

RNA extraction, library construction and sequencing

The total RNA samples were extracted using an E.Z.N.A. Bacterial RNA Kit (Omega, Bio-tek, Norcross, GA, USA), according to the manufacturer'sprotocol. The isolated RNA was digested with DNaseI (Ambion, Carlsbad, CA, USA) to remove possible traces of DNA. The concentration of the total RNA was determined with a spectrophotometer, and its quality was assessed on a 1 % agarose gel. Multiple 30-μg quantities of each RNA sample were depleted of rRNA using a Ribo-Zero™ rRNA

Removal kit (Epicenter, USA) according to the manufacturer's instructions. The resulting RNA samples were dissolved in 100 μL RNase-free water and quantified with a NanoDrop 2000 spectrophotometer (Wilmington, DE, USA).

The mRNA samples were incubated with biotinylated random hexamers (Illumina, San Diego, CA, USA) with the use of 1,000 units of Superscript II reverse transcriptase (Invitrogen, Carlsbad, CA, USA) for first-strand cDNA synthesis. Dynal M280 streptavidin Dynabeads (Invitrogen) were used to select the biotinylated RNA/cDNA. The first-strand of cDNA was released via alkaline hydrolysis. Subsequently, adaptors were ligated to the 5'-end of the first strand cDNA by DNA ligase (TaKaRa, Otsu, Japan), and the second-strand cDNA was synthesized through primer extension using ExTaq polymerase (TaKaRa, Japan). The synthesized cDNA was fractioned ultrasonically into 300–800 bp and purified with Ampure beads (Agencourt, USA). The prepared cDNAs were transformed into libraries using the Truseq™ DNA Sample Prep Kit-Set A (Illumina) and then clonally amplified with the TruSeq PE Cluster Kit (Illumina). DNA sequencing was performed on a HiSeq 2500 sequencing system (Illumina) by the Beijing Genomics Institute (BGI, China).

Real-time PCR analysis of gene expression

The transcriptome data validation and the levels of mRNA expressed by the wild-type strain *(wt),* the Al_2O_3 NPs-, and bulk Al_2O_3-treated strains were assessed using quantitative real-time PCR. First strand cDNA was synthesized using the RNA samples taken at 60 min, 12 h, and 24 h and the Thermoscript RT System (Invitrogen, Paisley, UK). PCR reactions were performed using the SYBR Green Real Time PCR

Master Mix (ABI, USA) according to the manufacturer's protocol in a StepOne Plus (ABI, USA). The internal reference genes were rrsB and MetK, encoding the 16s rRNA subunit and S-adenosylmethionine synthase, respectively, selected through the transcriptome analysis. All the primers used are listed in Table S1.

References

1. Kaweeteerawat, C.; Chang, C. H.; Roy, K. R.; Liu, R.; Li, R. B.; Toso, D.; Fischer, H.; Ivask, A.; Ji, Z. X.; Zink, J. I.; Zhou, Z. H.; Chanfreau, G. F.; Telesca, D.; Cohen, Y.; Holden, P. A.; Nel, A. E.; Godwin, H. A., Cu Nanoparticles Have Different Impacts in Escherichia coli and Lactobacillus brevis than Their Microsized and Ionic Analogues. *Acs Nano* **2015,** *9*, (7), 7215-7225.
